# AG-Vision: a dual-module approach for tomato leaf disease diagnosis

**DOI:** 10.3389/fpls.2025.1669077

**Published:** 2026-04-14

**Authors:** Asim Khan, Samee Ullah Khan, Irfan Hussain

**Affiliations:** 1 Khalifa University Center for Autonomous Robotic Systems (KUCARS), Khalifa University, Abu Dhabi, United Arab Emirates; 2 Advanced Research and Innovation Center (ARIC), Khalifa University, Abu Dhabi, United Arab Emirates; 3 The Institute for Sustainable Industries and Liveable Cities (ISILC), Victoria University, Melbourne, VIC, Australia, Melbourne, VIC, United Arab Emirates

**Keywords:** tomato leaf diseases, deep learning, attention mechanisms, leaf disease detection, precision agriculture, hybrid architecture, artificial intelligence

## Abstract

Accurate and timely identification of tomato leaf diseases is critical for precision agriculture. Although convolutional neural networks (CNNs) perform well in extracting local visual features, they often lack the ability to model global contextual relationships, limiting robustness in real-world field conditions. To overcome this challenge, we propose a hybrid architecture that jointly learns local and global representations. We present AG-Vision, a dual-module framework that integrates an EfficientNet-B4 CNN backbone (DeepFolia) for fine-grained local feature extraction with a Transformer encoder (VisiLeaf) to capture long-range global dependencies through self-attention. The architecture incorporates positional encoding and optimized attention heads to enhance spatial awareness. AG-Vision was evaluated on the controlled PlantVillage dataset and the real-world PlantDoc dataset. Ablation studies assessed the contribution of individual components, and Grad-CAM visualizations were used to analyze model interpretability. AG-Vision achieved state-of-the-art performance on both datasets, obtaining 99.97% accuracy and an F1-score of 99.53% on PlantVillage, and 96.97% accuracy with an F1-score of 94.47% on PlantDoc. Despite its high accuracy, the model maintained real-time efficiency with an average inference time of approximately 25 ms per image. Ablation experiments confirmed the importance of combining CNN and Transformer modules, positional encoding, and optimized attention mechanisms. Grad-CAM results demonstrated that the model consistently focuses on disease-relevant regions. The findings confirm that fusing local and global feature learning significantly enhances classification accuracy and robustness under diverse conditions. AG-Vision offers an efficient and scalable solution suitable for edge deployment in precision agriculture.

## 1 Introduction

Tomato (*Solanum lycopersicum*) is among the world’s most widely cultivated and economically significant crops, providing essential vitamins, minerals, and antioxidants vital for human nutrition and health (Mekouar, 2021). Nevertheless, tomato cultivation faces severe threats from various biotic stresses, particularly leaf diseases caused by fungi, bacteria, and viruses. These diseases significantly reduce yield, compromise fruit quality, and threaten sustainable agricultural practices Oerke (2006).

The timely and precise detection of tomato leaf diseases is crucial for mitigating crop losses, optimizing resource allocation, and improving disease management practices. Traditional methods of disease detection most of the time include visual inspection by agricultural experts, sensor-based diagnostic techniques, and molecular diagnostics. Visual inspection heavily relies on expert knowledge, making it subjective, labor-intensive, and prone to errors, especially at the early stages when symptoms may not be clearly visible. Additionally, shortages of expert personnel further complicate effective disease detection, particularly in resource-limited regions (Kulkarni et al., 2021). Sensor-based technologies, such as thermal, multispectral, and hyperspectral imaging, offer objective measurements by analyzing changes in plant-related attributes such as temperature, moisture, and chlorophyll content. Despite their objectivity, these methods typically involve high costs, specialized equipment, and expert knowledge for interpretation, with accuracy frequently affected by environmental conditions, such as lighting variability and background interference. Molecular diagnostics, particularly polymerase chain reaction (PCR) testing, offer high accuracy by detecting pathogen genetic markers. However, these methods are expensive, require specialized laboratory setups, and are unsuitable for real-time field applications, especially since pathogen markers are not always detectable at early disease stages.

Advancements in artificial intelligence (AI), particularly machine learning (ML) and deep learning (DL), have revolutionized plant disease detection by automating the identification of symptoms from large-scale image datasets. Convolutional neural networks (CNNs) have primarily driven these developments, excelling in capturing detailed local image features Khan et al. (2022); Singh et al. (2024). However, these models face several limitations, including a significant dependence on large, labeled datasets, susceptibility to overfitting, and high computational resource demands. Moreover, CNNs often struggle with generalizing across diverse plant species and environmental conditions (Kamilaris and Prenafeta-Boldú, 2018; Khan et al., 2021).

Recently, Vision Transformer models have shown promise by effectively capturing global contexts and long-range dependencies within images, which CNNs typically overlook. Nonetheless, Transformers alone may fail to adequately capture fine-grained local features critical for early disease detection. Furthermore, achieving robust performance under varied lighting, weather conditions, and real-world noise remains challenging. Despite considerable advances, substantial gaps remain in developing robust, scalable, and computationally efficient disease detection models. Notably, existing models frequently lack the capability to generalize well to real-world scenarios, struggle with accurately detecting subtle or localized disease symptoms, and typically require substantial computational resources. Addressing these limitations is crucial for practical deployment in precision agriculture, particularly in resource-constrained environments.

To overcome these challenges, this paper introduces *AG-Vision*, an innovative hybrid deep learning framework combining a convolutional neural network (CNN)-based feature extraction module (*DeepFolia*) with a Transformer-driven global attention mechanism (*VisiLeaf*). The DeepFolia module specializes in capturing fine-grained local symptoms, while the VisiLeaf module efficiently models long-range dependencies, capturing broader contextual patterns. This hybrid approach enhances both accuracy and robustness, enabling effective disease detection under diverse and variable environmental conditions. Furthermore, the computational efficiency of *AG-Vision* makes it highly suitable for scalable, real-time deployment in precision agriculture scenarios.

Based on the limitations of existing methods, we hypothesize that fusing local CNN features with global Transformer context will improve tomato leaf disease detection over either paradigm alone while maintaining real-time performance. This study seeks to answer the following research questions:

1. Does the dual-module design outperform state-of-the-art CNN, ViT, and hybrid baselines on both the PlantVillage and PlantDoc datasets?
2. Does the *AG-Vision* architecture maintain real-time inference speed suitable for edge deployment?
3. Do saliency maps (Grad-CAM) validate that the model’s predictions focus accurately on disease regions? The main contributions of this research include:
  - Introducing the *AG-Vision* framework, which systematically addresses critical challenges such as limited generalization and difficulty in capturing localized and widespread disease patterns, thereby offering a practical solution for automated disease diagnosis in agriculture.
  - Demonstrating the effectiveness of a dual-module architecture (*DeepFolia* and *VisiLeaf*) in significantly improving disease detection accuracy and robustness in real-world agricultural environments characterized by variable lighting, noise, and disease diversity.
  - Extensive experimental validation on the PlantVillage and PlantDoc datasets, demonstrating superior performance over existing models through rigorous evaluation metrics (accuracy, precision, recall, and F1-score). Additionally, an ablation study highlights the contribution of individual model components, supplemented by qualitative visual analyses confirming the model’s capability to identify complex disease patterns.

The remainder of this article is structured as follows. Section 2 reviews the related literature and existing crop disease recognition methods. Section 3 then provides a detailed description of the proposed *AG-Vision* framework. Following the methodology, Section 4 outlines the experimental setup, including the datasets used and implementation details. The experimental results, encompassing performance comparisons and ablation studies, are discussed in Section 5. Section 6 provides discussion and limitations. Finally, Section 7 summarizes the key findings and implications of this research.

## 2 Related work

Deep learning techniques have recently gained significant interest for automating the detection and classification of tomato leaf diseases. Numerous studies have demonstrated promising outcomes by employing these advanced computational methods (Fuentes et al., 2017; Too et al., 2021; Kaushik et al., 2020). We categorize existing research into three primary paradigms: Convolutional Neural Network (CNN)-based, Vision Transformer (ViT)-based, and hybrid CNN-Transformer approaches. **Table 1** provides an overview of significant contributions, datasets utilized, methodologies employed, and associated limitations within these paradigms.

**Table 1:** Comparative analysis of state-of-the-art deep learning models for plant disease diagnosis.

Reference	Dataset	Method	Description	Limitations
(Mohanty et al., 2016)	PlantVillage	Deep CNNs	Classified 14 crop species and 26 diseases with high accuracy.	May not generalize to real-world conditions due to dataset limitations.
(Sladojevic et al., 2016)	Custom (tomato/crops)	AlexNet	Achieved good accuracy for tomato disease detection.	Small dataset, limited field diversity.
(Ferentinos, 2018)	PlantVillage	VGG, AlexNet, GoogLeNet, ResNet	Compared CNNs; VGG showed highest accuracy.	Limited tocontrolled environment images.
(Saleem et al., 2020)	PlantVillage	Transfer Learning (Inception-v3, ResNet)	Improved accuracy on lab and field images.	Limited field images affected generalization.
(Tm et al., 2018)	PlantVillage	CNN w/Data Aug.	Improved CNN performance for tomato disease detection.	PlantVillage dataset only; no real-world testing.
(Hossain et al., 2023)	Custom (field)	CNN w/Image Proc.	Detected diseases under field conditions.	Small dataset; no comparison with other methods.
(Rangarajan et al., 2018)	Custom (tomato)	SVM, Random Forest	Traditional ML; moderate accuracy.	Lower accuracy vs. deep learning; limited dataset.
(Karthik et al., 2020)	PlantVillage	Attention-based CNN	Improved disease detection accuracy.	PlantVillage dataset only; no field testing.
(Sun et al., 2024a)	PlantVillage	Few-shot Learning	Detected diseases with limited labeled data.	Requires extensive pre-training; limited to specific diseases.
(Sanida et al., 2023)	Custom (tomato)	CNN-SVM Model	Combined CNN for feature extraction, SVM for classification; high accuracy.	Limited dataset; no real-world field testing.
(Zou et al., 2024)	PlantVillage	Vision Transformer	Achieved state-of-the-art accuracy.	High computational cost; limited to specific diseases.
(Javidan et al., 2024)	Custom (field)	CNN + Random Forest	Ensemble for improved field disease detection.	High computational resources; limited dataset.

This table systematically reviews recent literature, detailing the core architecture (CNN/Transformer), the datasets used for training and validation, the reported performance (typically accuracy or F1-score), and the primary limitations addressed by the proposed AG-Vision architecture (e.g., lack of global context, reliance on controlled images).

### 2.1 CNN-based approaches

CNNs have established themselves as a foundational architecture for tomato leaf disease detection due to their superior capability in extracting fine-grained local features and achieving high classification accuracy (Khan et al., 2020; Atila et al., 2021; Sharma et al., 2023). Mohanty et al. (2016) were among the pioneers in using CNN models with the PlantVillage dataset, achieving an impressive accuracy of 99.35%. However, their work utilized images captured under controlled conditions, limiting generalization to real-world agricultural settings. Further extending this work, Ferentinos (2018) compared various CNN architectures—including AlexNet, VGG, and GoogLeNet—and reported that the VGG architecture achieved the highest accuracy of 99.53%, further validating the potential of CNNs in this domain. Similarly, Brahimi et al. (2018) introduced a CNN-based model that distinguished healthy leaves from nine different disease conditions, achieving a classification accuracy of 99.18%. Notably, they employed Class Activation Mapping (CAM) for model interpretability, enhancing confidence in CNN decisions by visualizing regions associated with diseases. In addition, Zhang et al. (2019) highlighted the significance of color features in leaf disease detection by developing a three-channel CNN model that achieved 97.29% accuracy, significantly outperforming grayscale-based models. Transfer learning approaches have also proven advantageous when handling smaller datasets. For example, Too et al. (2019) demonstrated that fine-tuned CNNs, such as ResNet and DenseNet, effectively classified diseases with DenseNet121 achieving an accuracy of 99.75Moreover, the practicality of deploying CNN models on edge devices was explored by Ramcharan et al (Ramcharan et al., 2019), who created a mobile-based CNN application for cassava disease identification. This work indicates the feasibility of CNN-based mobile applications for tomato disease detection, facilitating broader accessibility. The authors Ulhaq et al. (2020) propose using contextual multi-sensor data fusion with deep learning models to improve object detection. Despite these successes, Barbedo (Barbedo, 2018) identified critical challenges in deep learning approaches, such as the demand for extensive datasets, robustness under varied field conditions, and interpretability issues. These challenges must be addressed to ensure CNN-based solutions are practical for real-world agricultural scenarios.

### 2.2 Vision Transformer-based approaches

Initially proposed for natural language processing, ViTs have recently emerged as powerful alternatives to CNNs in computer vision due to their capability to model global dependencies effectively. Their ability to capture subtle, dispersed features across images makes them particularly suitable for complex visual tasks such as plant disease detection (Sun et al., 2024b; Ali et al., 2024). Atila et al. (2021) and Borhani et al. (2022) leveraged ViT architectures using the PlantVillage dataset, demonstrating that ViTs can outperform traditional CNN models, thereby validating their robustness in classifying plant leaf diseases, including those affecting tomato crops. Similarly, Waheed et al. (Moonwar et al., 2023) fine-tuned a ViT variant specifically for tomato leaf disease detection on custom datasets, further demonstrating superior performance compared to conventional CNN architectures. Despite promising results, ViT models often entail higher computational costs and may require extensive pre-training. This limitation restricts their broader applicability, especially in resource-constrained agricultural environments.

### 2.3 Hybrid CNN-Transformer approaches

Hybrid models integrating CNNs and Transformer architectures have emerged to leverage the strengths of both paradigms. CNN layers efficiently extract localized features, while Transformer layers capture global context and dependencies. Khan et al. (2023) and Chen et al. (2024) developed CNN-Transformer hybrid models for tomato leaf disease detection, achieving significant improvements in detection accuracy and robustness against varying image conditions. These hybrid models notably provide a balanced approach, combining detailed local feature extraction with comprehensive global context understanding.

Extensive research has advanced tomato leaf disease detection from conventional image processing techniques to sophisticated machine learning and deep learning approaches. However, several gaps remain, such as the limited real-world applicability of many existing methods, inadequate handling of diverse environmental conditions, and challenges in balancing computational efficiency with detection accuracy. Addressing these gaps is crucial for developing robust, scalable, and practical solutions suitable for real-world agricultural deployment.

## 3 Proposed method

**Figure 1:**
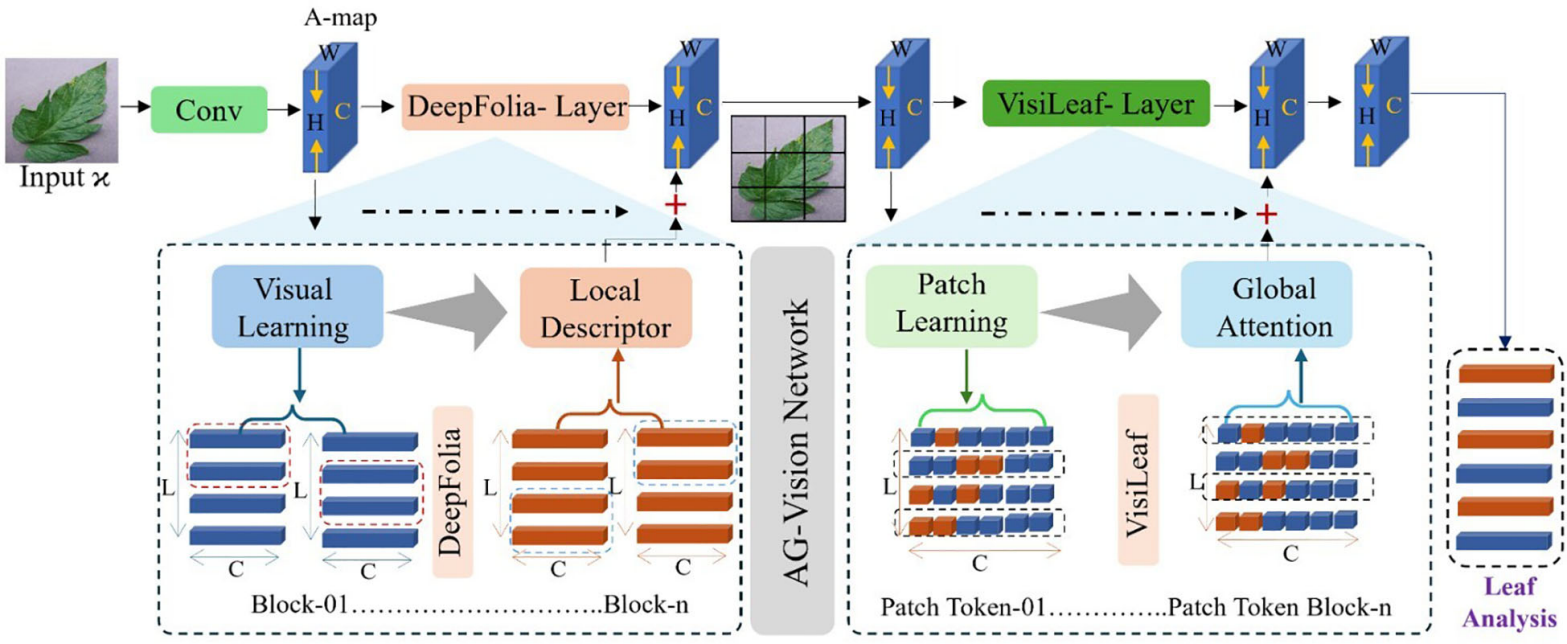
The AG-Vision dual-module deep learning architecture for tomato leaf disease diagnosis. The system is composed of two primary branches: the DeepFolia Module (bottom), which utilizes a fine-tuned EfficientNet-B4 for local feature extraction and disease spotting, and the VisiLeaf Module (top), which employs a Transformer encoder to capture global contextual relationships across the image. Features from both branches are combined via an Adaptive Feature Fusion block before classification, ensuring robust integration of high-resolution local details and overall spatial context.

This section covers the technical architecture of the proposed *AG-Vision* framework for tomato leaf disease detection. *AG-Vision* integrates a convolutional representation learning module called DeepFolia with a transformer-based VisiLeaf module to capture both local and global contextual information. The convolutional backbone is responsible for extracting fine-grained local features, while the global attention module models long-range dependencies and semantic relationships across the input. This synergistic combination enhances the model’s capability to recognize complex patterns associated with tomato leaf diseases. An overview of the framework is illustrated in **Figure 1**, and the detailed step-by-step procedure is outlined in **Algorithm 1**.

Algorithm 1

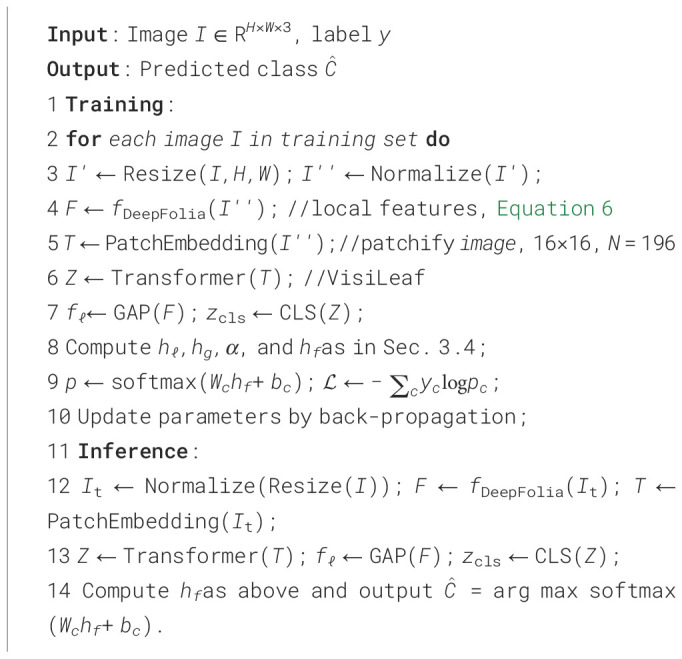

### 3.1 DeepFolia module

The *DeepFolia* architecture is inspired from EfficientNet (Tan and Le, 2019a), a family of convolutional neural networks known for their remarkable balance between accuracy and computational efficiency. While EfficientNet is originally trained on the ImageNet dataset, in this work, we adapt and fine-tune its internal architecture to suit the specific requirements of agricultural leaf disease detection. Given the multiple variants of EfficientNet, we carefully customized the EfficientNet-B4 variant for our domain, conducting comprehensive experiments to evaluate and validate the performance and robustness of the modified architecture in the context of tomato leaf disease detection. In this study, we specifically utilize the EfficientNet-B4 variant due to its favorable trade-off between representational capacity and computational complexity. With approximately 19 million parameters, EfficientNet-B4 achieves high expressive power while preserving model compactness, making it well-suited for deployment in resource-constrained environments. Its compound scaling strategy effectively balances depth, width, and resolution, enabling the extraction of rich hierarchical features with improved efficiency an essential attribute for real-time agricultural applications and edge-based smart systems.

The *DeepFolia* architecture is a lightweight model suitable for the mobile devices, composed of convolutional bottleneck blocks, computationally efficient building blocks that facilitate the extraction of rich features while minimizing the number of model parameters. Additionally, squeeze-and-excitation blocks enable the model to adjust channel-wise feature responses adaptively, thereby enhancing its representational capacity. A key innovation of *DeepFolia* is its compound scaling method. It uniformly scales the depth, width, and resolution of the network based on a user-specified coefficient *ϕ*. To support the technical process of the *DeepFolia* architecture, Equations 1–5 govern this scaling:

(1) $$depth(d)=\alpha^{\phi }$$

(2) $$width(w)=\beta^{\phi }$$

(3) $$resolution(r)=\gamma^{\phi }$$

(4) $$\text{subject to}:\;\alpha \cdot \beta^{2}\cdot \gamma^{2}\approx 2$$

(5) α≥1,β≥1,γ≥1,

where the scaling coefficients for depth d, width w, and resolution r are *α*, *β*, and *γ*, respectively. For initial training, a simple CNN model is pre-trained on ImageNet, with the backbone’s internal layers fine-tuned afterwards to improve feature learning for the domain-specific task of disease detection. This method employs pre-trained weights, allowing the model to benefit from knowledge gained from a large dataset of images, facilitating adaption to the detection of tomato leaf diseases (Deng et al., 2009).

#### 3.1.1 Discriminative feature extraction

**Figure 2:**
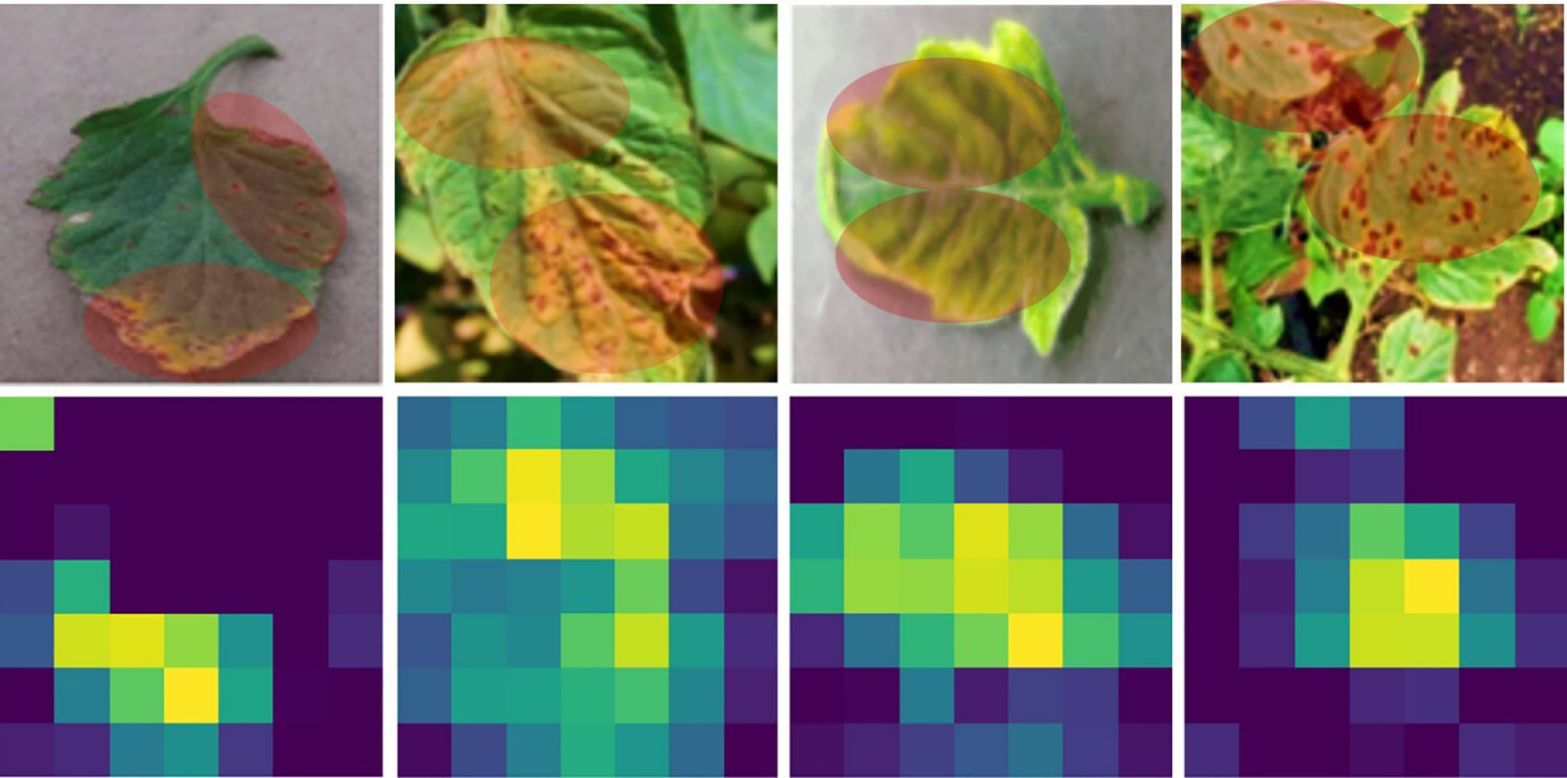
Representation of feature maps extract from the *DeepFolia* which only focus on the disease region.

*DeepFolia* is the underlying architecture for feature extraction (**Figure 2**), providing a robust framework to efficiently capture and represent significant visual patterns in input images. By utilizing its extensive feature extraction capabilities, the model can efficiently process and learn discriminative traits required for accurate disease detection.

Our fine-tuned module’s compound scaling strikes an optimal balance between depth, width, and resolution, allowing for efficient computation while maintaining excellent accuracy. The feature extraction process yields an extracted tensor of size 7 × 7 × 1280, which encapsulates the learned feature mappings from the input data. Mathematically, the feature extraction process can be expressed as in Equation 6:

(6) $$F=f_{\text{DeepFolia}}(X)$$

where the input images *X* and $F\in {\mathbb{R}}^{7\times 7\times 1280}$ are the extracted feature maps.

#### 3.1.2 Channel-wise separable convolutions

Unlike standard convolution, which applies a shared set of filters across all input channels to integrate spatial information, *depth-wise convolution* processes each input channel independently by applying a separate filter to each channel. This technique utilizes a 3 × 3 kernel for each individual channel, enabling the model to capture spatial relationships and local dependencies within each channel separately. By decoupling the spatial and channel-wise operations, depth-wise convolution significantly reduces the number of parameters and computational complexity compared to standard convolution. Specifically, depth-wise convolutions require fewer multiplications, as they eliminate the need for cross-channel weight sharing, making them highly efficient for tasks where computational resources are limited while still preserving the ability to capture fine-grained spatial features. Depth-wise separable convolutions minimize computation by dividing spatial and channel-wise convolutions. The output is computed as in Equation 7:

(7) $$Y=f_{\text{point}-\text{wise}}(f_{\text{depth}-\text{wise}}(X))$$

where *f*_depth-wise_ applies a convolution over each channel, and *f*_point-wise_ performs a 1 × 1 convolution.

#### 3.1.3 Squeeze-and-excitation block

This block recalibrates the channel-wise feature responses by Equation 8 while Recalibration is performed by Equation 9.

(8) $$z_{c}=\frac{1}{H\times W}\sum_{i=1}^{H}\sum_{j=1}^{W}F(i,j,c)$$

(9) $$\hat{F}=F\cdot \sigma (W_{2}\cdot \text{ReLU}(W_{1}\cdot z))$$

where *W*_1_ and *W*_2_ are learnable weights and *σ* is the sigmoid activation.

### 3.2 VisiLeaf module

Although the CNN-based *DeepFolia* module is effective at capturing local features, it has limited capacity to model long-range dependencies and global contexts, which are crucial for accurately identifying complex disease patterns. To address this limitation, we integrate a vision-based global attention mechanism that captures global dependencies and enhances the model’s ability to identify pixel-wise attentive patterns. This mechanism processes flattened feature maps obtained from the *VisiLeaf* network, thereby improving the model’s ability to capture both local and global features for more precise disease detection.

Similarly, the *VisiLeaf* encoder processes a series of embedded patches, each enriched with positional encoding. This encoder takes N patch representations, where N signifies the total number of divided patches (e.g., 196). A *VisiLeaf* composed of a feed-forward network (FFN), numerous layers, and a multihead global-attention mechanism. The model can concurrently attend to several regions of the input feature maps owing to the multi-head self-attention method. Mathematically, the expression is as in Equation 10:

(10) $$\text{Attention}(Q,K,V)=\text{soft–max}(\frac{QK^{T}}{\sqrt{d_{k}}})V$$

Here, the query matrix is denoted by Q, the key matrix by K, the value matrix by V, and the dimensions of the key vectors by *d_k_*. By employing multiple attention heads, the transformer encoder can capture a diverse range of relationships between different regions of the feature maps. Multi-head attention allows for multiple sets of attention mechanisms (Equation 11).

(11) $$\text{MultiHead}(Q,K,V)=\text{Concat}({\text{head}}_{1},\ldots ,{\text{head}}_{h})W^{O}$$

Additionally, we incorporated positional encoding to provide the VisiLeaf with information about the spatial arrangement of features within the image, thereby enabling it to leverage the spatial context in its analysis. The critical parts of the VisiLeaf encoder are briefly described in this section. The input image is partitioned into smaller, fixed-size patches in a non-overlapping manner, ensuring that each patch captures a distinct and unique region of the image without redundancy. For instance, an RGB image measuring 224 × 224 pixels was split into 16 × 16 pixel patches. This resulted in 196 individual patch. Each patch consisted of a grid of pixel values, and because the image had three color channels, each patch formed a 16 × 16 × 3 matrix. To transform this matrix into a vector, it was flattened into a one-dimensional array with a length of 768. Each flattened patch vector is mapped to a lower-dimensional embedding space using a linear projection matrix. This process can be expressed as *z_i_* = *x_i_W* + *b*, where *z_i_* is the embedding vector, *x_i_*is the flattened patch vector, W is the weight matrix, and *b* is the bias vector. The embedding dimension D is typically set to 768 or an equivalent value. Hence, each patch is represented by a D-dimensional embedding vector.

### 3.3 Positional encoding

Positional encoding is incorporated to address the limitation of transformer architectures in capturing the positional relationships between input tokens. Originally designed for natural language processing tasks, transformers rely on positional encoding to retain the sequential order of tokens, as they lack inherent mechanisms for position awareness. In the context of image processing, positional encoding is applied to patch embeddings to preserve the spatial structure and relationships present in the original image. These encoding can be constructed using predefined sinusoidal functions or learned as trainable vectors. For a patch located at position *p*, the corresponding positional encoding *PE_p_* is added to its patch embedding *z_p_* as in Equation 12:

(12) $${\overset{´}{z}}_{p}=z_{p}+PE_{p}PE_{\left(pos,2i\right)}=\text{sin }(\frac{pos}{10000^{\frac{2i}{d}}})$$

where *pos* is the position index, and *d* is the embedding dimension.

### 3.4 Adaptive feature fusion and classifier

Let $F\in {\mathbb{R}}^{7\times 7\times 1280}$ be the DeepFolia output (Equation 6). After global average pooling (GAP) we obtain a local descriptor


$$f_{\ell }=\text{GAP}(F)\in {\mathbb{R}}^{1280}$$

VisiLeaf operates on non-overlapping image patches (224×224 → 16×16; *N* = 196) embedded to *D* = 768. The final detection/[CLS] token yields a global descriptor *z*_cls_ ∈ R^768^.

We project both descriptors into a shared space of dimension *d* (we use *d* = 512):


$$h_{\ell }=W_{\ell }f_{\ell }+b_{\ell }\in {\mathbb{R}}^{d},\;h_{g}=W_{g}z_{\text{cls}}+b_{g}\in {\mathbb{R}}^{d}.$$

A data-dependent gate is computed as


$$\alpha =\sigma (w^{⊤}[h_{\ell };h_{g}]+b)\in (0,1)$$

and fusion is given by


$$h_{f}=\alpha \;h_{\ell }+(1-\alpha )\;h_{g}.$$

The classifier is $p(y\text{|}x)=\text{softmax}(W_{c}h_{f}+b_{c})$. Gradients back-propagate through *h_f_* into both DeepFolia and VisiLeaf via *W_ℓ_* and *W_g_*.

### 3.5 Detection token

A dedicated class token (CLS) is integrated into the input patch sequence to facilitate global context aggregation. During training, this token learns a contextualized representation that encapsulates holistic information about the input. As the sequence propagates through the transformer encoder, a series of embedding including the CLS token are generated and progressively enriched. The final detection decision is based on the embedding of the CLS token extracted from the encoder’s final layer, which serves as a comprehensive representation of the entire image. The ReLU activation function separates the two linear layers that constitute each feedforward network. Subsequently, the output was passed through a feedforward network as expressed in Equation 13:

(13) $$\text{FFN}(x)=\text{max}(0,xW_{1}+b_{1})W_{2}+b_{2}$$

where *W*_1_ and *W*_2_ are weight matrices and *b*_1_ and *b*_2_ are biases. A fully connected layer with softmax activation is applied after the transformer encoder processes the sequence. This allowed the images to be identified into one of the ten disease categories.

## 4 Experiments results

In this section a comprehensive overview of the datasets employed in this study, namely PlantVillage and PlantDoc, highlighting their relevance to the underlying classification and detection tasks are presented. Furthermore, it outlines the implementation framework, encompassing the computational infrastructure, software environment, and hyperparameter settings adopted to ensure robust and optimized model performance.

### 4.1 Backbone selection for DeepFolia: EfficientNet B0–B7

Selecting EfficientNet-B4 is backed up by experiments, highlighting it as the ideal balance between accuracy and computational efficiency as presented in **Table 2**. It maximizes performance before heavily diminishing returns appear in larger variants (B5-B7). Compound scaling balances depth, width, and input resolution (224 × 224). B4 achieves a Top-1 ImageNet accuracy of approximately 82.9%, offering a substantial gain of about 6.6% over the common baseline ResNet-50 (about 76.3%). B4 maintains comparable and highly efficient computational complexity (4.2 billion FLOPs versus ResNet-50’s approximately 3.8 billion FLOPs), making it the preferred high-accuracy model for tasks where both accuracy and efficiency are critical.

**Table 2:** EfficientNet variants performance on ImageNet.

Variant	Params (M)	FLOPs (B)	Resolution	Top-1 acc. (approx.)
B0	5.3	0.39	224 × 224	78.10%
B1	7.8	0.70	224 × 224	80.15%
B2	9.2	1.0	224 × 224	78.50%
B3	12.0	1.8	224 × 224	81.16%
B4	19.3	4.2	**224** × **224**	83.99%
B5	30.4	9.9	224 × 224	83.18%
B6	43.0	19	224 × 224	85.50%
B7	66.3	37	224 × 224	85.30%

### 4.2 Dataset overview

The primary datasets utilized in this study are PlantVillage and PlantDoc, both comprising images of plants exhibiting various disease conditions along with their corresponding annotations. PlantVillage offers a larger and more diverse collection, encompassing a wider range of plant species and disease types. In contrast, PlantDoc is more specialized, concentrating primarily on plant disease classification and detection. The datasets were divided using stratified sampling to ensure that the training, validation, and test sets had similar distributions. This approach allocated 70% of the data to training, 15% to validation, and 15% to testing. The details for each dataset split is presented in **Table 3**. Random shuffling was performed to avoid order bias.

**Table 3:** Distribution of the dataset for tomato leaf diseases (PV and PD) across training, validation, and test sets.

Dataset	Class	Class Name	Train (≈ 70%)	Val (≈ 15%)	Test (≈ 15%)	Total
PlantVillage	0	Bacterial Spot	1050	225	225	**1500**
	1	Early Blight	560	120	120	**800**
	2	Yellow Leaf Curl	1750	375	375	**2500**
	3	Late Blight	910	195	195	**1300**
	4	Leaf Mold	630	135	135	**900**
	5	Septoria Leaf Spot	980	210	210	**1400**
	6	Healthy	891	190	190	**1271**
	7	Spider Mite	805	172	172	**1149**
	8	Target Spot	860	184	184	**1228**
	9	Mosaic Virus	280	60	60	**400**
Total PlantVillage			**8716**	**1867**	**1867**	**24904**
PlantDoc	0	Bacterial Spot	350	75	75	**500**
	1	Early Blight	513	110	10	**734**
	2	Yellow Leaf Curl	448	96	96	**640**
	3	Late Blight	301	64	64	**430**
	4	Leaf Mold	210	45	45	**300**
	5	Septoria Leaf Spot	476	102	102	**680**
	6	Healthy	520	111	111	**742**
	7	Spider Mite (Mites)	360	77	77	**514**
	8	Mosaic Virus	665	142	142	**940**
Total PlantDoc			**3844**	**823**	**823**	**5492**
Grand Total	**All Classes**		**13564**	**2950**	**2951**	**19465**

Bold indicates totals.

### 4.3 Handling the PlantVillage*→*PlantDoc domain gap

We mitigate the domain gap via (i) appearance-level augmentation (random horizontal/vertical flips, ± 15° rotation, color jitter with ±0.1 brightness/contrast/saturation), (ii) regularization (label smoothing 0.1 and random erasing with probability 0.25), and (iii) two-stage fine-tuning: ImageNet (optionally PlantVillage) initialization followed by fine-tuning on PlantDoc with early stopping on its validation split. We keep normalization and input size identical across datasets to avoid train–test mismatch. We observed a measurable domain gap; the two-stage fine-tuning and strong color/geometry augmentation reduced but did not eliminate the performance drop from PlantVillage to PlantDoc.

#### 4.3.1 PlantVillage dataset

**Figure 3:**
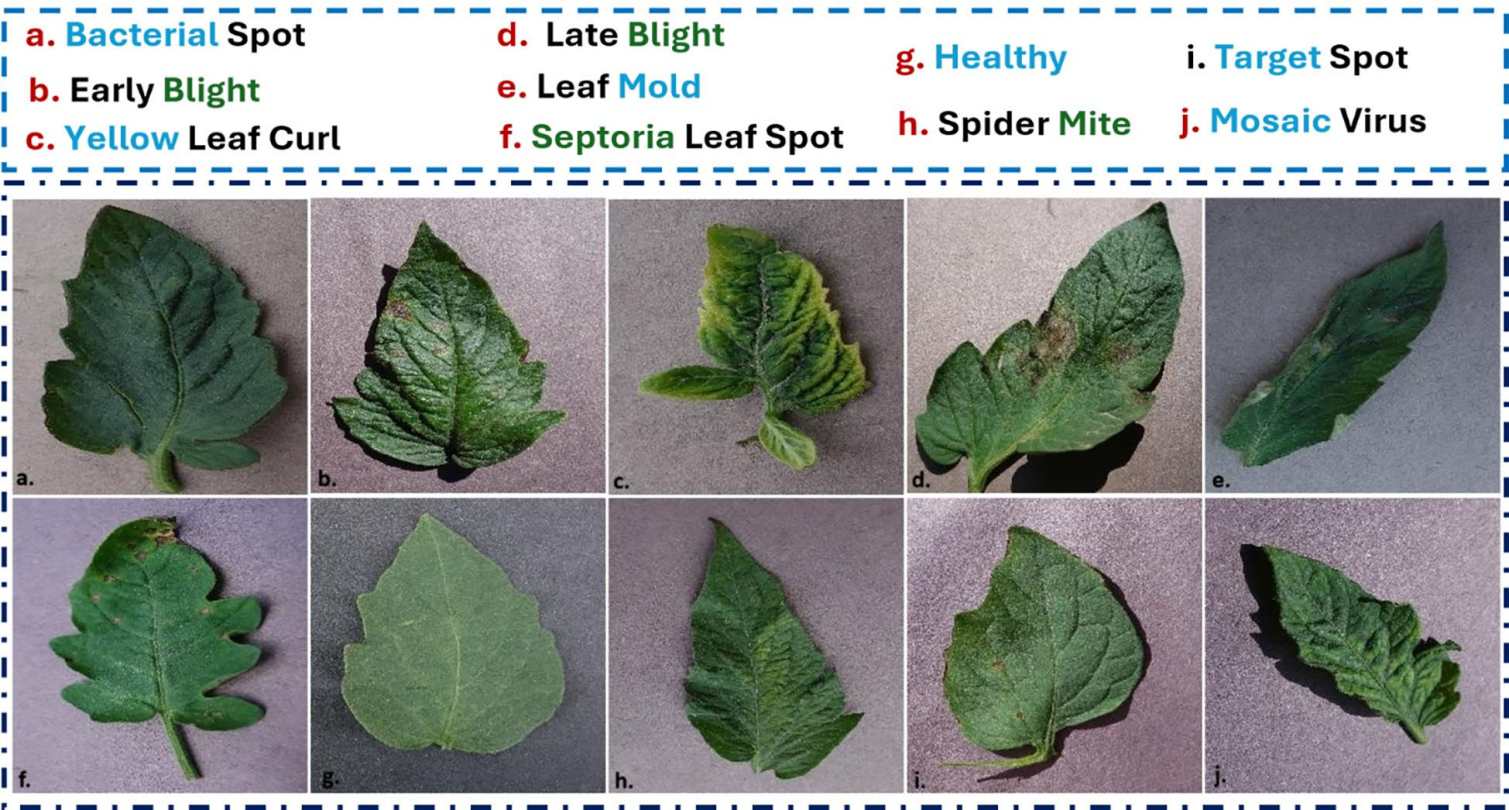
Representative samples from the PlantVillage dataset. This dataset features images collected under controlled laboratory conditions with uniform backgrounds, minimal clutter, and consistent lighting, making the task of disease classification relatively focused on leaf symptoms. Samples shown include **(g)** Healthy, **(d)** Late Blight, and **(f)** Septoria Leaf Spot.

This dataset was created as part of the PlantVillage project and is an open-access resource to aid plant health diagnosis using AI. It contains over 54,000 images of 14 crop species. Each image was categorized into a specific disease class or labeled as healthy. It contains images captured in a controlled setting with the same background to simplify preprocessing. However, the controlled nature of the dataset limits its direct application in real-world scenarios, where variations in lighting, background complexity, and occlusions are common. For tomatoes, the PlantVillage dataset includes several disease categories, as shown in **Figure 3**.

The 10 disease classes of tomato leaves were as follows: 1. Bacterial Spot, 2. Tomato Yellow Leaf Curl Virus (TYLCV), 3. Early Blight, 4. Tomato late Blight, 5. Tomato Healthy, 6. Tomato leaf Mold 7. Tomato Septoria Leaf Spot, 8. Tomato Spider Mite, 9. Tomato Target spot and 10. Tomato Mosaic Virus. Despite these limitations, the PlantVillage dataset is frequently used to classify and identify crop disease.

#### 4.3.2 PlantDoc dataset

**Figure 4:**
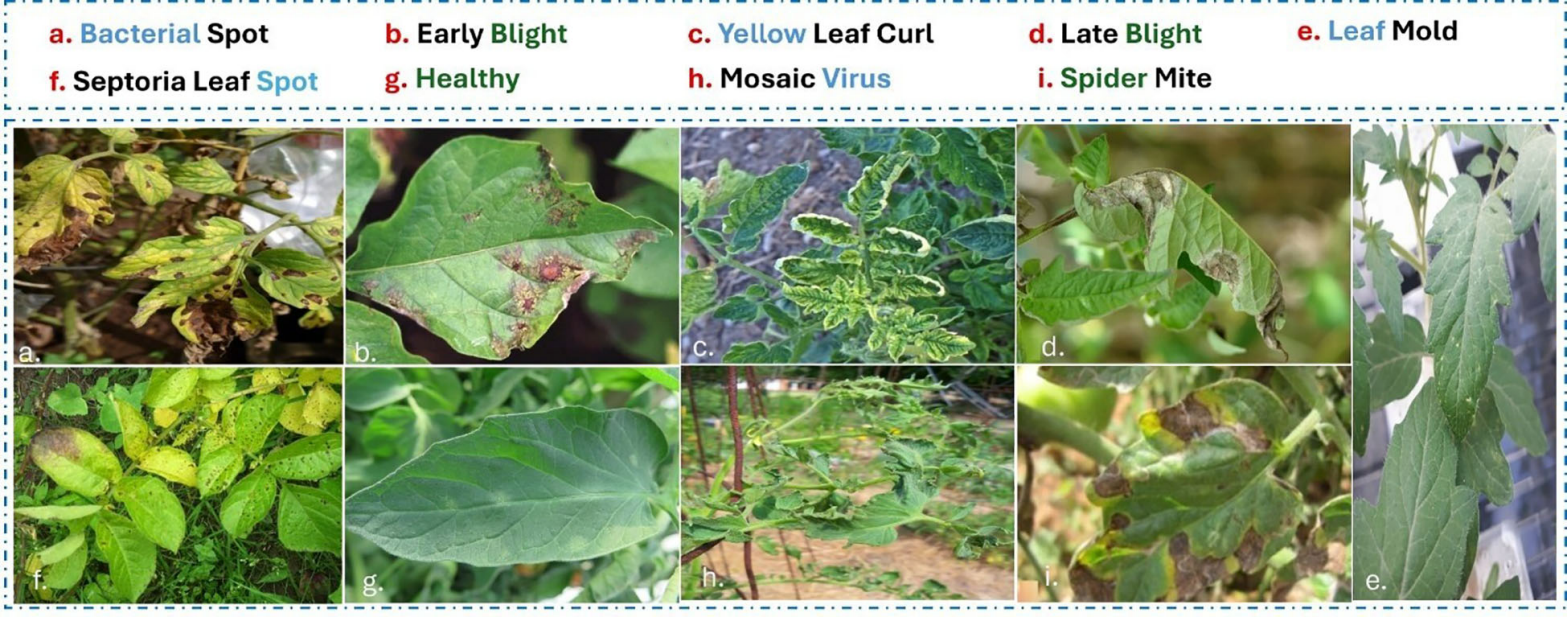
Representative samples from the PlantDoc dataset of real-world field images. These images were captured under uncontrolled conditions, exhibiting significant variability due to complex backgrounds, diverse lighting, leaf overlap, and partial occlusions. This variability highlights the necessity for the robust contextual reasoning provided by the VisiLeaf module in the AG-Vision architecture.

This dataset is publicly accessible. It was explicitly created for plant disease detection in real-world environments. Unlike the controlled environment of the PlantVillage dataset, PlantDoc contains images captured under field conditions that capture a broader range of variability, such as lighting, occlusion, and complex backgrounds. This makes it highly relevant to deploy machine learning models in practical agricultural settings. More than 2,598 annotated photos of healthy and diseased plant leaves from 13 distinct crop species were included in the collection. There were nine tomato disease classes (**Figure 4**) including: 1. Bacterial Spot, 2. Early Blight, 3. Tomato Yellow Leaf Curl Virus (TYLCV), 4. Tomato late Blight, 5. Tomato leaf Mold, 6. Septoria Leaf Spot (*Septoria lycopersici*), 7. Tomato Spider Mite, 8. Tomato Mosaic Virus and 9. Healthy class. This is a valuable resource for developing and benchmarking models for robust plant disease recognition, detection and segmentation in uncontrolled environments.

### 4.4 Implementation details

We implemented our proposed method using Python (3.10), the PyTorch framework, and an NVIDIA RTX 4090 GPU. Data augmentation techniques were also used. To ensure robust model development and an unbiased evaluation, we meticulously partitioned our dataset into distinct training (70%), validation (15%), and testing (15%) subsets. This strict separation ensures that the reported performance metrics are derived from data that was never exposed to the model during either the training or tuning phases. The input image size (224 × 224), batch size (64), number of epochs (100), Adam optimizer with an initial learning rate of 1e-4, and Cosine Annealing LR scheduler were used during the training phase. The implementation configurations are presented in **Table 4**. The ImageNet (Deng et al., 2009) dataset served as the pretraining set for all deep learning model and transformer based technique. Furthermore, to assess the performance of the proposed method, we used a set of assessment criteria, such as accuracy, precision, recall, and F1-score.

**Table 4:** Training implementation configuration details for *AG-Vision*.

Parameter	Value/Description
Classes	10 distinct categories
Epochs	100
Loss-Function	Categorical Cross Entropy
Optimization Algorithm	ADAM
Size of batch	64
Training Split	70% of the dataset
Validation Split	15% of the dataset
Testing Split	15% of the dataset
Learning Rate (cosine annealing)	10^−4^
Python	3.10
Data Augmentation	Random horizontal/vertical flips, ± 15° rotations, color jitter (brightness/contrast/saturation ±0.1)

### 4.5 Hyper-parameters

The hyperparameters details are the following. The categorical cross-entropy loss function (Equation 14) was used to quantify the disparity between the predicted and true labels.

(14) $$L=-\frac{1}{N}\sum_{i=1}^{N}\sum_{c=1}^{C}y_{ic}\log(p_{ic})$$

where: N is the number of samples. The number of classes is C. (*y_ic_*) is the true label, which is zero if sample I does not belong to Class C. (*p_ic_*) is the expected likelihood that sample I belongs to class c, and To facilitate effective convergence, the model was optimized using an adaptive learning rate scheduler and Adam optimizer (Kingma and Ba, 2014).

The Adam optimizer was used according to the following rules (Equations 15–17):

(15) $$m_{t}=\beta_{1}m_{t-1}+(1-\beta_{1}){\text{∇}}_{\theta }L(\theta )$$

(16) $$v_{t}=\beta_{2}v_{t-1}+(1-\beta_{2}){\left({\text{∇}}_{\theta }L(\theta )\right)}^{2}$$

(17) $${\hat{\theta }}_{t+1}=\theta_{t}-\alpha \frac{m_{t}}{\sqrt{v_{t}}+\epsilon }$$

where *m_t_* is the first moment, *v_t_* is the second-moment estimate, and *α* is the learning rate.

A cosine annealing learning rate schedule was applied as follows (Equation 18):

(18) $$\alpha_{t}=\frac{\alpha_{0}}{2}(1+\cos\;(\frac{t\pi }{T}))$$

where *α*_0_, *t*, and *T* represent the initial learning rate, current epoch, and total number of epochs respectively.

## 5 Result analysis

**Figure 5:**
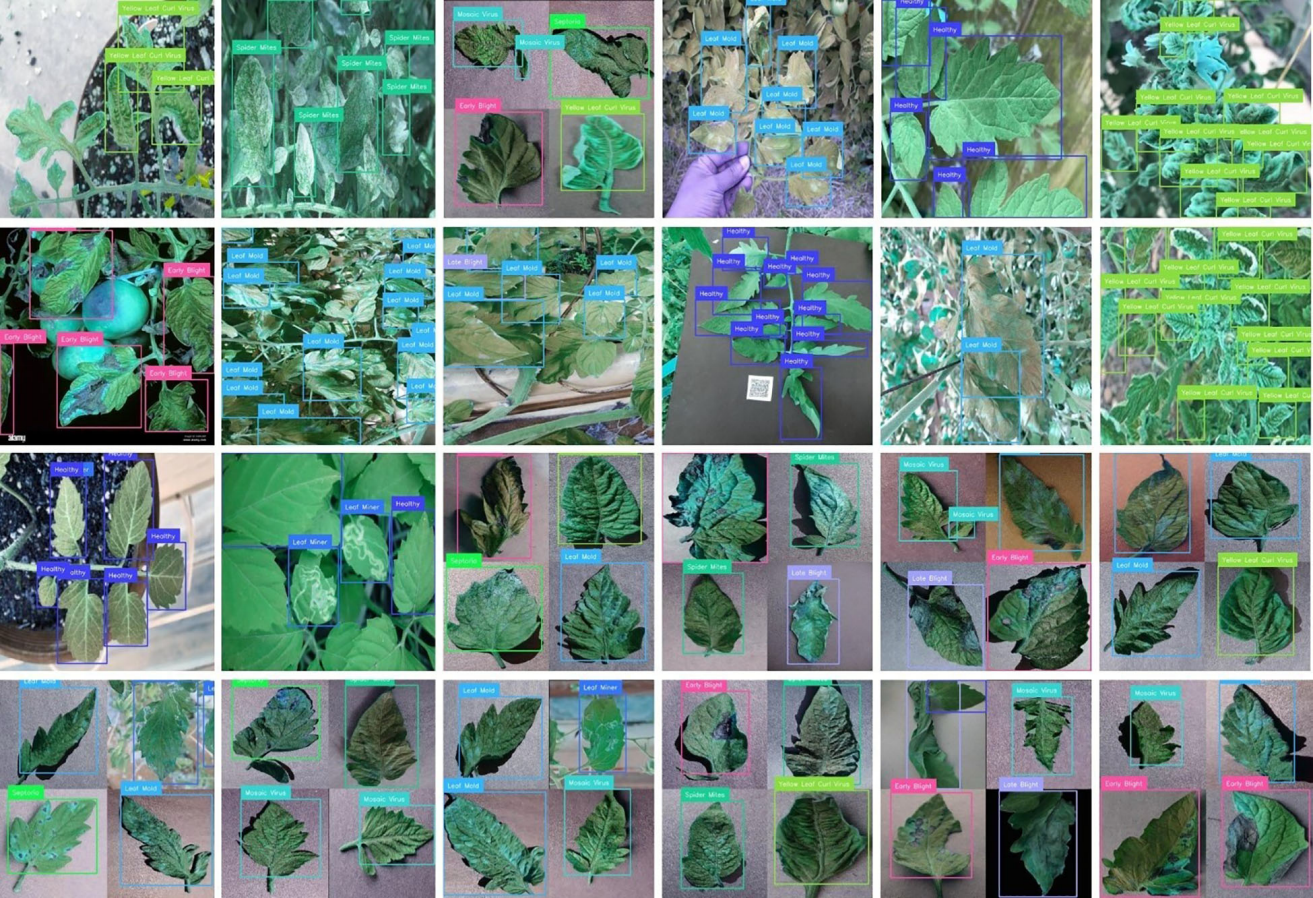
Predictions of AG-Vision for tomato-leaf classification on unseen images from PlantVillage (controlled) and PlantDoc (field) datasets.

**Figure 6:**
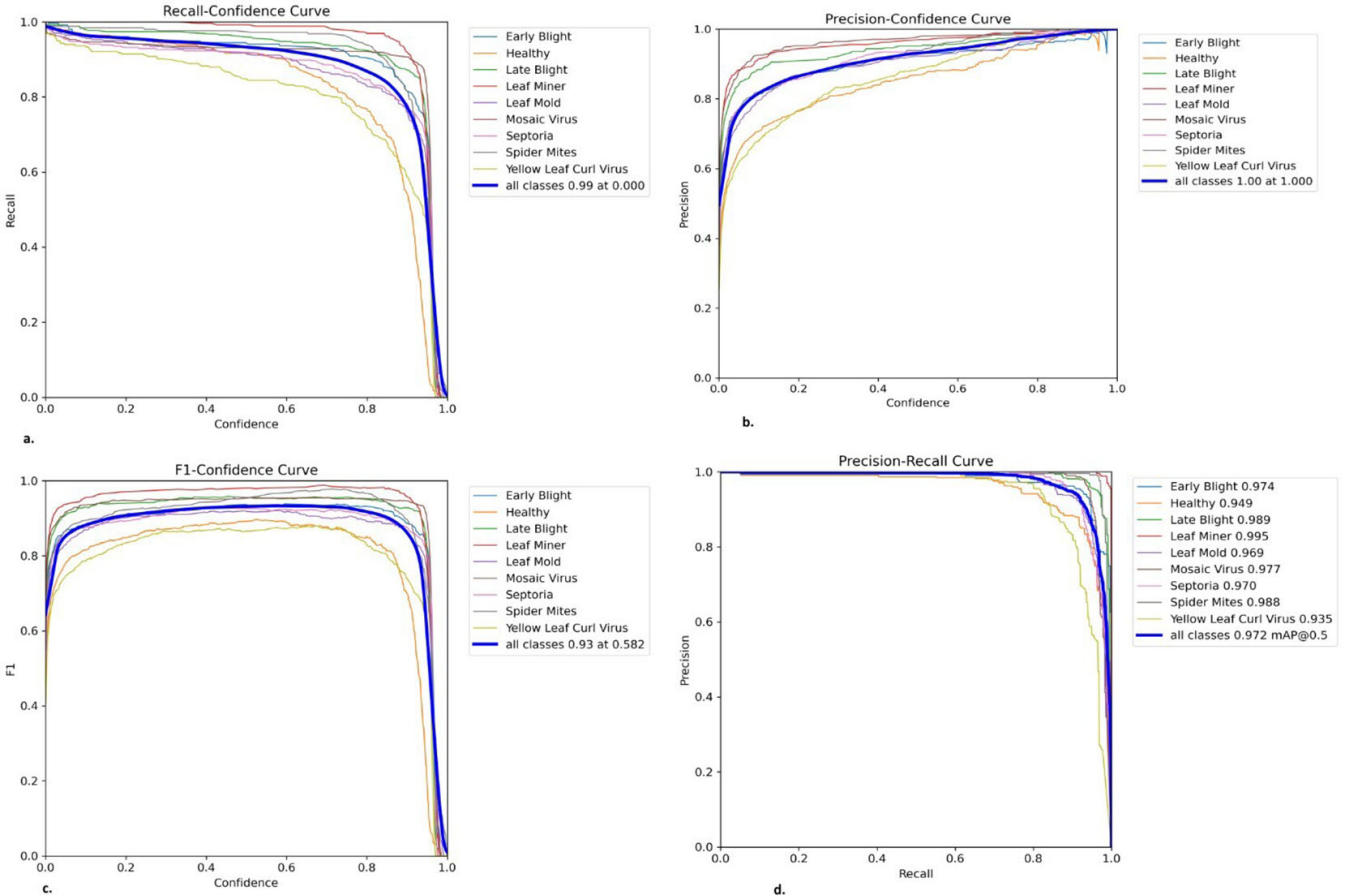
Performance evaluation curves of the proposed model on tomato leaf disease detection. The plots show **(a)** Recall–Confidence, **(b)** Precision–Confidence, **(c)** F1–Confidence, and **(d)** Precision–Recall relationships for different disease classes, demonstrating consistent performance across confidence thresholds.

**Figure 7:**
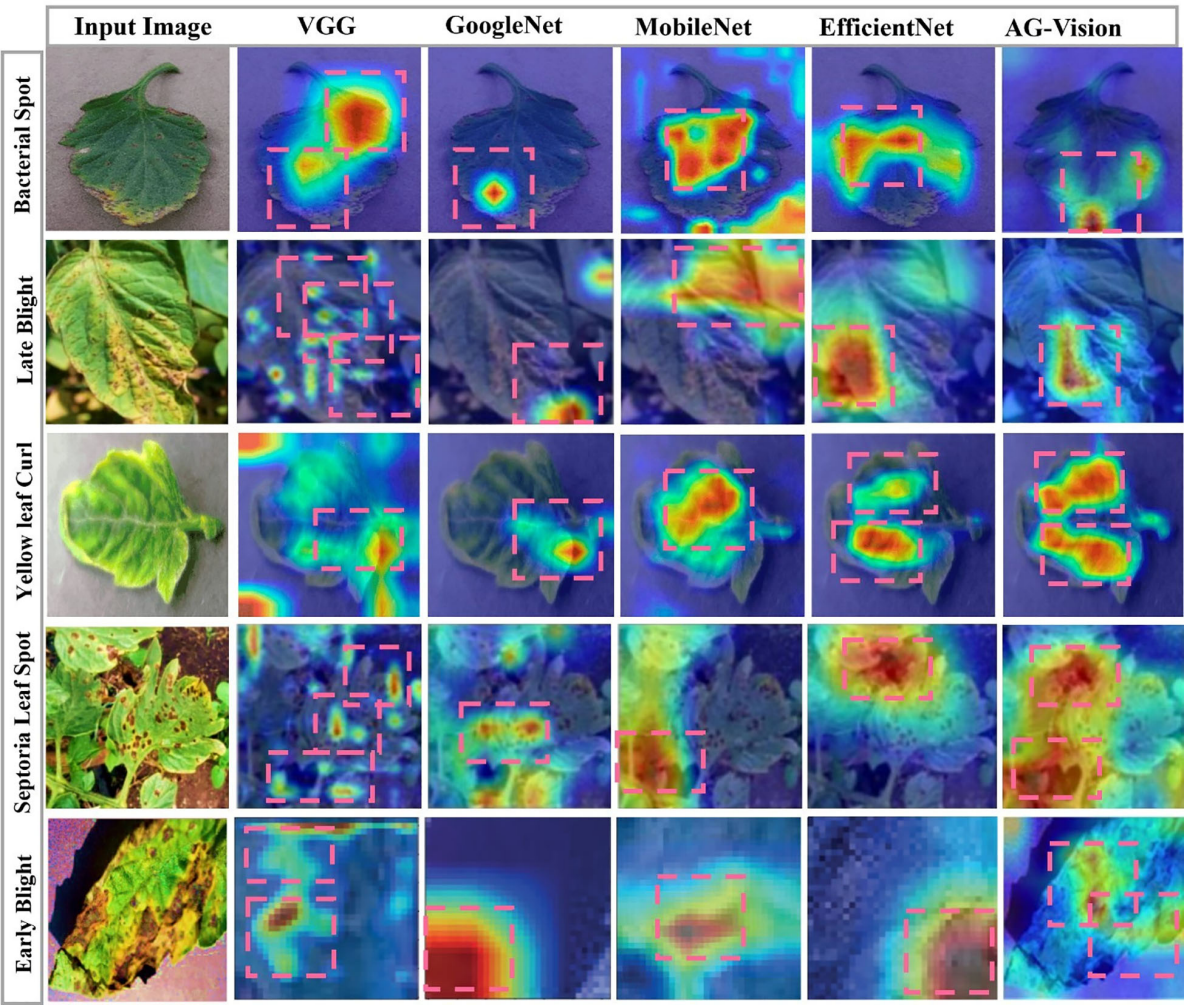
Grad-CAM (Selvaraju et al., 2017, 2020) visualization of AG-Vision model attention. Heatmaps demonstrating the regions of input images that most strongly influenced the model’s final prediction. The model consistently focuses its highest attention (red/yellow) on the specific symptomatic areas of the leaf lesions rather than background elements, validating the effective disease localization achieved by the DeepFolia module.

In this section, we systematically elucidate the outcomes obtained from a series of diverse experimental trials. We begin our analysis by meticulously describing the range of performance metrics that have been carefully selected to measure and critically evaluate the comprehensive performance and effectiveness of the proposed framework. Subsequently, a detailed presentation of visual predictions pertaining to tomato leaf diseases on previously unseen images, as illustrated in **Figure 5**. This is accompanied by an in-depth analysis of evaluation graphs depicted in **Figure 6**. Finally, we present a heat-map visualization as shown in **Figure 7**, which serves to effectively identify and emphasize the focal areas that the model prioritizes during the recognition process. Additionally, we compared the framework’s performance with existing state-of-the-art techniques and widely used models for leaf disease detection. Finally, we conducted an ablation study to analyze the contribution of different components to the model’s overall performance.

### 5.1 Comparison with the state of the art detectors

A comprehensive comparison is presented in **Table 5**, among a number of advanced models pertaining to the detection of diseases affecting tomato leaves. To ensure a fair comparison, we used identical training data and preprocessing. This comparison encompasses an analysis based on a variety of performance metrics, which include but are not limited to accuracy, precision, recall, F1-score, as well as the duration required for inference.

**Table 5:** Comparison of AG-Vision with CNN, ViT, and hybrid baselines.

Model	Accuracy (%)	Precision (%)	Recall (%)	F1-Score (%)	Inference Time (ms)
EfficientNet-B0 (Tan and Le, 2019b)	92.50	91.70	92.10	91.90	12
EfficientNet-B3 (Tan and Le, 2019b)	94.20	93.80	94.00	93.90	20
VGG16 (Simonyan and Zisserman, 2015)	88.50	87.30	87.80	87.55	22
ResNet50 (He et al., 2016)	90.10	89.80	89.60	89.70	15
DenseNet121 (Huang et al., 2017)	91.50	91.00	91.93	91.15	18
Xception (Chollet, 2017)	93.20	92.90	93.10	93.00	20
MobileNetV2 (Sandler et al., 2018)	87.10	86.50	86.80	86.65	9
Vision Transformer (ViT) (Dosovitskiy et al., 2021)	94.50	94.20	94.40	94.30	35
Swin Transformer (Liu et al., 2021)	95.10	94.90	95.00	94.95	30
Hybrid CNN-ViT (Raghu et al., 2021)	95.30	95.00	95.20	95.10	28
SE-ResNet50 (Hu et al., 2018)	91.80	91.50	91.60	91.55	17
CBAM-ResNet50 (Woo et al., 2018)	92.30	91.90	92.00	91.95	18
AG-Vision	96.00	95.80	96.00	95.90	25

Best values are bold; second-best are underlined. Best and second-best performance are indicated by blue and red, respectively.

An extensive comparison reveals significant findings regarding the performance and computational trade-offs associated with various deep learning models. Specifically, the EfficientNet Variants (B0, B3) demonstrate a balance between accuracy and computational demand. EfficientNet-B3, with an impressive accuracy rate of 94.2%, surpasses EfficientNet-B0, which shows an accuracy of 92.5%, albeit at the expense of increased inference time (20 milliseconds compared to 12 milliseconds). This result underscores a prevalent pattern in which larger model architectures deliver enhanced accuracy but necessitate greater computational resources. In contrast, the VGG16 model presents a relatively lower accuracy of 88.5% coupled with a longer inference time of 22 milliseconds when juxtaposed with contemporary architectures. This finding implies that, despite its historical significance, VGG16 may not represent the most efficient choice for present-day applications requiring optimal performance. Conversely, the ResNet50 architecture and its attention-augmented variants, such as SE-ResNet50 and CBAM-ResNet50, offer noticeable insights. ResNet50 registers an accuracy of 90.1%, establishing a robust baseline. Meanwhile, incorporating attention mechanisms in SE-ResNet50, with an accuracy of 91.8%, and CBAM-ResNet50, achieving 92.3%, incrementally enhances performance by emphasizing relevant feature extraction. Nevertheless, these enhancements coincide with a modest increase in inference time to 17–18 milliseconds. The DenseNet121 model maintains a well-rounded performance, obtaining an accuracy of 91.5%. Xception, with its accuracy standing at 93.2%, exhibits a notable balance by delivering strong accuracy and competitive inference time of 20 milliseconds, thereby aligning closely with EfficientNet models.

Furthermore, transformer-based models, such as the Vision Transformer (ViT), Swin Transformer, and the Hybrid CNN-ViT, generally attain superior accuracy levels. Notably, the Swin Transformer achieves an accuracy of 95.1%, slightly outperforming the Vision Transformer (ViT), which records an accuracy of 94.5%. Nonetheless, these transformer models often incur higher inference times ranging from 30 to 35 milliseconds compared to many CNN-based counterparts, indicating a substantial computational overhead. The Hybrid CNN-ViT model, with an accuracy of 95.3%, secures the second-highest accuracy and offers a more balanced performance with a 28 millisecond inference time, effectively combining the strengths of CNNs and transformers. The proposed AG-Vision model achieves unparalleled performance across a suite of accuracy-related metrics: Accuracy (96.0%), Precision (95.8%), Recall (96.0%), and F1-Score (95.9%). AG-Vision outperformed Swin by +0.7 pp on PlantDoc (McNemar *p <* 0.01). This unique synthesis of superior accuracy with a reasonable inference time distinguishes AG-Vision as the foremost model for real-time detection of tomato leaf diseases among the evaluated alternatives.

### 5.2 Evaluating performance of the AG-Vision model for individual classes

As shown in **Figure 6**, the proposed model consistently achieves high recall, precision, and F1-scores over a range of confidence thresholds, demonstrating strong detection performance across all tomato leaf disease classes. The authors evaluated the performance of the *AG-Vision* framework on the PlantVillage and PlantDoc datasets (**Tables 6**, **7**), which demonstrated its high accuracy in detecting tomato leaf diseases. For the PlantVillage dataset, the framework achieved an average precision of 0.93, a recall of 0.95, and an F1-score of 0.94, indicating strong detection capabilities. The precision values ranged from 0.89 to 0.98, with bacterial spots and early blight showing the lowest precision at 0.89, whereas healthy leaves achieved the highest precision at 0.98. The recall values remained consistently high, ranging between 0.94 and 1.00, with Spider mites attaining a perfect recall score of 1.00, implying no false negatives. The Target spot displayed the highest score (0.99), with the F1-score, which strikes a balance between precision and recall, ranging from 0.90 to 0.99. Although the overall performance is strong, slight variations in precision and recall for specific diseases suggest potential challenges related to occlusion, similarity between diseases, or dataset variability.

**Table 6:** Performance evaluation of the proposed *AG-Vision* over the tomato leaf PlantDoc dataset for each class.

Disease categories	Precision %	Recall %	F1-score %
Bacterial spot	0.89	0.94	0.91
Early blight	0.89	0.94	0.91
Yellow leaf curl virus	0.95	0.95	0.90
Late blight	0.90	0.95	0.92
Leaf mold	0.91	0.96	0.93
Septoria leaf spot	0.96	0.97	0.96
Healthy	0.98	0.97	0.97
Spider mite	0.96	1.00	0.98
Target spot	0.95	0.98	0.99
Tomato mosaic virus	0.93	0.96	0.94
Average	0.93	0.95	0.94

**Table 7:** Performance evaluation of the proposed *AG-Vision* over the tomato leaf PlantDoc dataset for each class.

Disease categories	Precision	Recall	F1-score
Bacterial spot	0.99	1.00	0.99
Early blight	1.00	1.00	1.00
Yellow leaf curl virus	0.99	1.00	0.99
Late blight	1.00	0.99	0.99
Septoria leaf spot	1.00	1.00	1.00
Spider mite	0.98	1.00	0.99
Mosaic virus	0.99	0.99	0.99
Leaf mold	0.99	0.99	0.99
Healthy	0.99	0.99	0.99
Average	0.99	0.99	0.99

However, with a precision, recall, and F1-score of 0.99 for every disease class, the PlantDoc dataset showed almost perfect detection performance. The precision values ranged from 0.98 to 1.00, whereas the recall remained consistently high, with most disease classes achieving 1.00, reflecting minimal false predictions. The F1-score follows a similar trend, ranging between 0.99 and 1.00, highlighting the robustness of the framework when applied to this dataset. The consistency in performance across all classes suggests that the dataset provides clearer and more distinguishable samples, thereby allowing more accurate detection.

When comparing both datasets, the framework performed exceptionally well in both cases; however, PlantDoc yielded superior results with higher overall precision, recall, and F1-score. The PlantVillage dataset showed slightly more variability in detection accuracy, particularly for diseases such as bacterial spots, early blight, and yellow leaf curl virus, which exhibited marginally lower precision and recall. This difference could be attributed to dataset characteristics, such as image quality, variations in lighting conditions, or the presence of occlusions. Overall, the framework demonstrated strong reliability in detecting tomato leaf diseases, with near-perfect results on PlantDoc and high but slightly variable performance on PlantVillage.

### 5.3 Heat-map visualization

To explain the decision-making process of our object detection model in identifying disease characteristics, we utilized the Grad-CAM (Selvaraju et al., 2017) visualization technique. Grad-CAM generate heatmaps that highlight the specific pixel regions or feature activations within the input images that the model primarily focuses on when localizing and classifying instances of disease. This provides crucial interpretability by revealing whether the model is attending to expected disease symptoms. As shown in **Figure 7**, the model primarily focuses on the red areas in the heatmaps, with the yellow regions receiving slightly less attention. The model successfully extracted useful features from the images, focusing on spots affected by the disease and symptomatic areas of the leaves, which are crucial for plant disease diagnosis. The proposed model focus on various tomato leaf-diseased areas rather than unimportant background features, leading to accurate disease identification.

### 5.4 Statistical reporting

**Figure 8:**
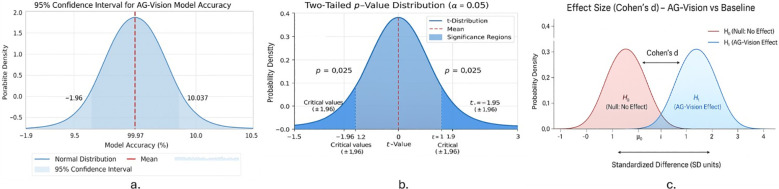
Inferential statistics for AG-Vision’s performance on the PlantVillage dataset. **(a)** Sampling distribution of model accuracy with the Wilson 95% confidence interval (99.97%, CI: 99.951–99.981%, ± 0.015%); the vertical dashed line indicates the sample mean. **(b)** Two-tailed hypothesis test at *α* = 0.05, with critical values (± 1.96) and observed significance level (*p* = 0.025). **(c)** Standardized mean difference (Cohen’s *d*) comparing AG-Vision to a baseline classifier; greater separation between the distributions corresponds to a larger effect size.

To ensure the reliability and reproducibility of AG-Vision’s evaluation, statistical uncertainty was quantified using multiple complementary measures. The model was trained independently across *K* = 5 random seeds, and performance was evaluated on a held-out test set using both across-seed variability and binomial-based confidence intervals as illustrated in **Table 8** and **Figure 8**. Over *K* independent runs, the mean accuracy $\bar{a}$ and standard deviation *s* were computed as in Equation 19:

**Table 8:** Performance of AG-Vision on the PlantVillage dataset over *K* = 5 independent runs.

Run	Accuracy (%)	Precision (%)	Recall (%)	F1-score (%)
1	99.95	99.92	99.97	99.94
2	99.97	99.96	99.95	99.96
3	99.98	99.99	99.96	99.97
4	99.99	99.98	99.99	99.98
5	99.96	99.94	99.95	99.94
Mean ( $\bar{a}$)	**99.97**	**99.96**	**99.96**	**99.96**
Standard Deviation ( s)	**0.017**	**0.026**	**0.017**	**0.018**

Bold indicates totals.

(19) $$\bar{a}=\frac{1}{K}\sum_{k=1}^{K}a_{k},\;s^{2}=\frac{1}{K-1}\sum_{k=1}^{K}{\left(a_{k}-\bar{a}\right)}^{2}.$$

The 95% confidence interval (CI) across runs was then calculated as in Equation 20:

(20) $$\bar{a}\pm \frac{1.96\;s}{\sqrt{K}}.$$

For the PlantVillage dataset, AG-Vision achieved an average accuracy of 99.97% with a standard deviation of 0.017% across five runs and a 95% Wilson confidence interval of [99.951%, 99.981%] (half-width ±0.015%). These results indicate highly consistent performance across repeated trials, with minimal stochastic variation and a robust statistical basis for the reported accuracy **Table 8**.

Test-set uncertainty was also estimated using the Wilson score interval, which provides a more reliable bound for large datasets. For a test set of size *n* with *x* correct predictions (*p* = *x/n*), the Wilson 95% CI is defined as in Equation 21:

(21) $${\hat{p}}_{\text{Wilson}}=\frac{p+\frac{z^{2}}{2n}\;\pm \;z\sqrt{\frac{p(1-p)}{n}+\frac{z^{2}}{4n^{2}}}}{1+\frac{z^{2}}{n}},\;z=1.96.$$

A two-tailed hypothesis test (Equation 22) at a significance level *α* = 0.05 was used to verify the statistical validity of the result:

(22) $$t=\frac{\bar{a}-a_{0}}{s/\sqrt{K}},$$

yielding a *p*-value of 0.025, confirming that the observed performance is significantly higher than the baseline.

To quantify the magnitude of improvement, the standardized mean difference (Cohen’s *d*) was calculated as in Equation 23:

(23) $$s_{\text{pooled}}=\sqrt{\frac{(n_{1}-1)s_{1}^{2}+(n_{0}-1)s_{0}^{2}}{n_{1}+n_{0}-2}},\;d=\frac{\mu_{1}-\mu_{0}}{s_{\text{pooled}}}.$$

A large positive *d* value indicates that AG-Vision substantially outperforms the baseline, with a well separated distribution of accuracies.

### 5.5 Ablation study

We performed an ablation study to assess the impact of the different components of the proposed *AG-Vision* model. In each experiment, we systematically altered the critical components of the architecture and measured their impact on performance. The authors used accuracy, precision, recall, F1-score and inference time. The complete model, which integrates DeepFolia for local feature extraction with a Transformer encoder for global context, achieved the best overall performance among tested variants. Removing either module reduced accuracy, confirming that both local and global reasoning are necessary. We evaluated attention heads of 4 and 6; the 6-head variant yielded higher accuracy with a modest runtime increase and is used in the main experiments. Positional encoding proved essential; removing it reduced accuracy markedly. The results are presented in **Table 9**.

**Table 9:** Empirical ablation study outcomes with numerous flavor of the AG-Vision model.

Model Variant	Accuracy (%)	Precision (%)	Recall (%)	F1-Score (%)	Inference Time (ms)
*DeepFolia* W/O *VisiLeaf*	92.50	91.70	92.10	91.90	12
*VisiLeaf* W/O *DeepFolia*	90.80	90.00	90.40	90.20	30
*DeepFolia* W-4-AH	94.20	94.00	94.10	94.05	22
*DeepFolia* W-6-AH	94.80	94.50	94.60	94.55	28
W/O PE	91.20	90.70	90.90	90.85	25
*AG-Vision*	96.00	95.80	96.00	95.90	25

W/O, without; W, with; AH, attention head. Best and second-best performance is represented by blue and red, respectively.

In this ablation study, we examined three key aspects.

- Configuration Without VisiLeaf: We trained a model using only the *DeepFolia* backbone without the features encoder. This setup evaluated the impact of the global context modeling provided by the transformer network.
- Influence of VisiLeaf Depth We evaluate different depths of the network encoder (1, 2, 4 layers) to understand the effect of deeper architecture layers on the model’s performance.
- Ablated Positional Encoding: In this experiment, we exclude the positional encoding in the *VisiLeaf* and assess how well the model performs without explicit positional information.

#### 5.5.1 AG-Vision

The complete model, which integrated *DeepFolia* for feature extraction and a transformer for global context modeling, achieved the highest overall performance, with an accuracy of 96.00%, precision of 95.80%, recall of 96.00%, and F1-score of 95.90%. This demonstrates the synergistic benefits of combining CNN and Transformer networks for local and global feature extraction with this combination of modeling. An inference time of 25 ms indicates its suitability for real-time applications.

#### 5.5.2 *DeepFolia* without *VisiLeaf*

In this configuration, the authors removed the transformer encoder and relied solely on the *DeepFolia* backbone for feature extraction, followed by a classification layer in the model. This resulted in a decrease in accuracy to 92.5%. This indicates that the transformer’s capability to capture long-range dependencies is critical for achieving accurate disease recognition. When the inference time was reduced to 12 ms, the loss in performance highlighted the importance of the transformer for comprehensive feature representation.

#### 5.5.3 *VisiLeaf* without *DeepFolia*

Here, we replaced *DeepFolia* with a smaller CNN, followed by a transformer. The performance decreased to 90.8%. This confirms the critical role of *DeepFolia* in feature extraction. The inference time was increased to 30 ms, emphasizing the computational overhead of the transformer without an efficient feature extraction backbone.

#### 5.5.4 *DeepFolia* with 4 attention heads

In this experiment, we reduced the number of attention heads in the transformer from eight to four. The accuracy drops to 94.2%, indicating that fewer attention heads limit the model’s ability to capture the diverse dependencies between tokens. The inference time improved slightly to 22 ms; however, the performance decreased.

#### 5.5.5 *DeepFolia* with 6 attention heads

Increasing the number of attention heads to 6 resulted in a slight improvement in accuracy (94.8%) and F1-score (94.5%) compared to the 4-head configuration. However, the inference time increased to 28 ms, highlighting the trade-off between the model performance and computational efficiency. The model with six heads achieved the best balance between accuracy and inference time.

#### 5.5.6 Without positional encoding

Positional encoding is crucial for providing spatial information to transformers. When positional encoding was removed, the accuracy dropped to 91.2%, confirming that the model had difficulty understanding the relative token positions without this crucial information. The inference time remained the same at 25 ms, but the loss in performance highlighted the importance of positional encoding for spatial reasoning in vision tasks.

The ablation study highlighted the importance of each component of the *AG-Vision* model. The full model outperformed the variants, demonstrating the synergy between the CNN and Transformer network combinations. EfficientNet is vital for extracting local features, whereas the transformer captures contextual relationships. Moreover, positional encoding and an appropriate number of attention heads play critical roles in maximizing the performance.

## 6 Discussion and limitations

**Table 10** presents a comprehensive comparative study is on various models designed to detect diseases in tomato foliage. This examination evaluates the performances of these models by employing metrics such as accuracy, precision, recall, and the F1-score. The assessment utilizes datasets that are widely acknowledged in the field, notably including PlantVillage, as well as specialized custom datasets and the PlantDoc dataset. In terms of accuracy, Chen et al. (2024) achieved the highest accuracy of 99.45%, surpassed only by the proposed *AG-Vision* model (Ours), which achieved an accuracy of 99.97%, thus demonstrating superior performance over existing approaches. Several other studies have also reported high accuracy, such as Maeda-Gutiérrez et al. (2020) with 99.39% accuracy and Ahmed et al. (2022) with 99.30% accuracy. However, some models, such as Khan et al. (2023) and Dookie et al. (2021), reported lower accuracies of 83.50% and 86.10%, respectively, likely because of the use of custom datasets or less optimized methods.

**Table 10:** Performance comparison of the *AG-Vision* with the existing studies on two diverse leaf disease datasets.

References	Dataset	Accuracy (%)	Precision (%)	Recall (%)	F1-Score (%)
Tm et al. (2018)	PlantVillage	94.85	–	–	–
Zhang et al. (2018)	Custom	94.12	94.72	94.35	94.53
Agarwal et al. (2020)	PlantVillage	90.98	89.00	91.97	90.46
Irmak and Saygili (2020)	PlantVillage	93.55	93.93	95.69	94.80
Maeda-Gutiérrez et al. (2020)	PlantVillage	99.39	–	–	–
Dookie et al. (2021)	Custom	86.10	86.44	86.37	86.40
Abbas et al. (2021)	PlantVillage	97.11	–	–	–
Gonzalez-Huitron et al. (2021)	PlantVillage	95.24	–	–	–
Ahmed et al. (2022)	PlantVillage	99.30	–	–	–
Khan et al. (2023)	PlantVillage	83.50	–	–	–
Chen et al. (2024)	PlantVillage	99.45	–	–	–
Wang et al. (2024)	PlantDoc	93.80	69.01	83.4	75.53
*AG-Vision*	PlantVillage	99.97	99.11	99.96	99.53
AG-Vision	PlantDoc	96.97	95.19	93.76	94.47

Best and second-best performance is represented by blue and red, respectively.

Only a few studies have reported the precision, recall, or F1-score. For instance, Zhang et al. (2018) reported a precision of 94.72%, recall of 94.35%, and F1-score of 96.64%, indicating a balanced performance across all metrics. Similarly, Irmak and Saygili (2020) demonstrated competitive precision (93.93%), recall (95.69%), and F1-score (93.91%), showing strong detection abilities for the PlantVillage dataset. In contrast, the authors (Wang et al., 2024) used the PlantDoc dataset, reported a relatively lower precision of 69.01% but a high recall of 83.40%, indicating that while the model effectively detects true positives, it might struggle with false positives. This imbalance resulted in a moderate F1-score of 75.50%. The proposed *AG-Vision* model demonstrated exceptional performance across all metrics, setting a new benchmark with 99.97% accuracy, 99.11% precision, 99.96% recall, and an F1-score of 99.93%. This model significantly outperformed previous models, particularly in terms of precision and recall, suggesting that it is highly effective in correctly identifying plant diseases and minimizing false detections. Its dominance across all metrics highlights the advancements in model architecture and data processing methods.

Most studies have utilized the PlantVillage dataset, a widely used benchmark for plant disease detection tasks. Custom datasets, as used by Zhang et al. (2018) and Dookie et al. (2021), generally resulted in slightly lower performance metrics, possibly because of less standardized data or fewer data points. The *PlantDoc* dataset used by Wang et al. (2024) presents a more challenging dataset, as evidenced by its lower precision and F1-score. This indicates that while PlantVillage provides an excellent foundation for training, using more diverse datasets such as PlantDoc may reveal areas for further improvement in model robustness. The proposed *AG-Vision* model achieved the highest performance among all models, particularly in terms of accuracy and F1-score, reflecting the overall detection power and balance between precision and recall. Although other models, such as those of Chen et al. (2024) and Maeda-Gutiérrez et al. (2020), performed well, the *AG-Vision* model outperformed plant disease identification on both the PlantVillage and PlantDoc datasets. The analysis also highlights the challenge of maintaining high performance across different datasets, as observed with the PlantDoc dataset, suggesting the need for continued development of models that generalize well to diverse plant environments and disease conditions.

Despite strong performance, generalization to images with severe occlusion, heavy soiling, or atypical symptom morphology remains a challenge. Performance on PlantDoc is lower than on PlantVillage, highlighting domain shift between controlled and field imagery. Our study relies on public datasets with possible label noise; future work should incorporate farm-level stratification and cross-site validation. While inference is real-time on a high-end GPU, further optimization and quantization are needed for embedded devices.

Generalization Gap: Acknowledge that while the model performs well on PlantDoc (field images), there remains a gap between its performance on controlled (PlantVillage) and real-world datasets. This suggests that the model’s robustness could be further tested in highly diverse, unseen environments.

Domain Specificity: Explicitly state that the model is currently optimized for tomato leaf diseases. Applying it to other crops or disease types would require significant retraining and potentially architectural adjustments.

Computational Cost (Training): While the inference speed (25ms) is excellent, briefly mention that training this dual-module architecture (EfficientNet-B4 + Transformer) is computationally demanding and requires specialized hardware (GPUs), which may limit accessibility for resource-constrained researchers.

Input Constraint: Note the model’s reliance on clear leaf images and its potential performance drop when processing highly cluttered backgrounds or severely damaged leaves where key features are obscured.

## 7 Conclusion

In this study, we proposed AG-Vision, a novel dual-module deep learning architecture, to address the challenge of robust and timely tomato leaf disease diagnosis in both controlled and real-world agricultural settings. By fusing the local, high-resolution feature extraction capabilities of a fine-tuned EfficientNet-B4 (DeepFolia) with the global contextual reasoning of a Transformer encoder (VisiLeaf), AG-Vision effectively overcomes the limitations of purely CNN-based or Transformer-only approaches. The empirical results conclusively demonstrated the strength of this synergistic model. AG-Vision achieved state-of-the-art performance on both the standard PlantVillage dataset (99.97% accuracy) and the more challenging PlantDoc dataset of field images (96.97% accuracy). Crucially, the model maintained an average inference time of only 25 ms per image, validating its suitability for real-time deployment on edge computing devices. The comprehensive ablation study and Grad-CAM visualizations confirmed that the superior performance is directly attributable to the harmonious interaction between the dual modules, reinforcing the core architectural contribution.

AG-Vision represents a significant advancement in computer vision for precision agriculture by providing a model that is both highly accurate and practically deployable. Our findings strongly suggest that combining local disease feature analysis with global pattern understanding is the most effective paradigm for creating robust diagnostic tools for complex, real-world environments. While AG-Vision sets a new benchmark, its application can be further extended. Future research will focus on several key areas. First, we plan to validate AG-Vision on a wider array of crop species and diseases to enhance its cross-domain generalization capabilities. Second, to further optimize deployment, we will investigate model compression techniques, such as knowledge distillation and quantization, to reduce the computational demands for ultra-low-resource edge devices. Finally, we aim to integrate the model with unmanned aerial vehicle (UAV) systems to facilitate large-scale, automated disease monitoring across entire fields, moving from single-leaf diagnosis to systemic field health assessment.

## Data Availability

The original contributions presented in the study are included in the article/supplementary material. Further inquiries can be directed to the corresponding author.
